# Noninvasive Detection of Oxidative Stress in a Mouse Model of 4R Tauopathy via Positron Emission Tomography with [^18^F]ROStrace

**DOI:** 10.3390/ijms26051845

**Published:** 2025-02-21

**Authors:** Evan Gallagher, Shihong Li, Hsiaoju Lee, Hong Xu, Virginia M.-Y. Lee, Robert H. Mach, Meagan J. McManus

**Affiliations:** 1 Department of Anesthesia and Critical Care Medicine, Children’s Hospital of Philadelphia, Philadelphia, PA 19104, USA; evangall@pennmedicine.upenn.edu; 2 Department of Radiology, University of Pennsylvania, Philadelphia, PA 19104, USArmach@pennmedicine.upenn.edu (R.H.M.); 3 Center for Neurodegenerative Disease Research, University of Pennsylvania, Philadelphia, PA 19104, USA

**Keywords:** tau, 4R tau, oxidative stress, reactive oxygen species, [^18^F]ROStrace, PET imaging, tauopathy, PS19, P301S

## Abstract

Oxidative stress, defined as the excessive production of reactive oxygen species (ROS), is a crucial factor in the pathogenesis of various neurodegenerative diseases, including the 4-repeat (4R) tauopathies. Collectively, the 4R tauopathies are characterized by the progressive aggregation of tau protein isoforms with four microtubule-binding domains in and around brain cells. The cyclical relationship between oxidative stress and 4R tau aggregation suggests that a means of imaging ROS noninvasively could be a valuable tool for the study and treatment of 4R tauopathy in both humans and animal models. To demonstrate the potential of the ROS-sensitive positron emission tomography (PET) radiotracer [^18^F]ROStrace as a means of filling this methodological gap, we performed [^18^F]ROStrace PET imaging on PS19 mice, which exhibit 4R tau aggregation similar to that seen in human 4R tauopathy. Significant increases in [^18^F]ROStrace signal became detectable in the hippocampus of 6–11-month-old (mo) PS19 animals and spread to the brainstem, midbrain, and thalamus of 11+ mo animals. Additionally, older PS19 mice displayed higher whole-brain average [^18^F]ROStrace signal compared to age-matched controls (*p* = 0.042), and tau pathology consistently colocalized with multiple fluorescent indicators of oxidative stress in PS19 brain samples. These results provide novel evidence that 4R tau aggregation is associated with increased oxidative stress in PS19 mouse brain and advance [^18^F]ROStrace as a noninvasive technology for the detection of oxidative stress in neurodegenerative diseases involving tau pathology.

## 1. Introduction

The 4-repeat (4R) tauopathies are a group of neurodegenerative disorders that are characterized by the abnormal aggregation of tau protein isoforms containing four microtubule-binding domains in and around brain cells. The most well-known 4R tauopathies are progressive supranuclear palsy (PSP) and corticobasal degeneration (CBD); however, 4R tau pathology is also observed in conditions such as argyrophilic grain disease (AGD), chronic traumatic encephalopathy (CTE), and Alzheimer’s disease (AD), among others [1]. Unfortunately, despite the considerable economic and societal burden imposed by the 4R tauopathies, outcome-altering interventions for these disorders remain elusive, with most current therapies providing temporary symptomatic relief but failing to alter the underlying course of the disease [2]. Consequently, there remains an urgent need for novel tools that can aid in the identification and treatment of 4R tauopathy, both in human patients and in preclinical models of disease.

A defining characteristic of all 4R tauopathies is the formation and deposition of insoluble 4R tau inclusions in and around neurons and glial cells. The fundamental processes that drive tau aggregation in the 4R tauopathies have not been fully elucidated; however, substantial evidence indicates that oxidative stress—i.e., detrimental overproduction of reactive oxygen species (ROS) relative to intracellular antioxidant defenses—is closely associated with aberrant tau aggregation. For example, brain tissue samples from human CBD [3], PSP [4], AGD [5], and AD [6,7,8,9,10] patients consistently show elevations in ROS-induced damage, indicating that oxidative stress likely represents a common pathological mechanism across all of these disorders. Likewise, age-dependent increases in oxidative stress have been reported in several animal models of tau aggregation [11,12,13], and multiple studies have demonstrated that oxidizing conditions promote tau hyperphosphorylation [14,15] and aggregation [16,17,18]. Notably, several animal studies have also reported that elevations in oxidative stress precede the formation of insoluble tau pathology [11,13], suggesting that imbalances in ROS production and clearance may occur relatively early in the disease process. Together, these findings clearly implicate oxidative stress as an important contributor to the pathogenesis of 4R tauopathy and suggest that ROS could potentially be leveraged as indirect biomarkers of tau aggregation in living animals. Unfortunately, the application of ROS as biomarkers is currently hindered by the absence of noninvasive methods for quantifying levels of ROS in vivo.

Positron emission tomography (PET) with the ROS-sensitive radiotracer [^18^F]ROStrace [19,20] has recently been used to detect elevations in ROS production in multiple rodent models of protein aggregation [21,22] and neurodegeneration [23], suggesting that [^18^F]ROStrace PET may also be capable of detecting 4R tau-associated oxidative stress in vivo. As described previously [20], [^18^F]ROStrace enters the brain as a neutral species and becomes trapped following oxidation by ROS, resulting in higher PET signal in brain regions with increased ROS production. Since 4R tau aggregation has previously been associated with increased ROS production in rodent brain [11,12,13], we hypothesized that the average brain [^18^F]ROStrace signal would be higher in mice displaying 4R tau aggregation compared to age-matched healthy controls. Further, we expected tau-associated increases in [^18^F]ROStrace signal to become larger and more widespread within the brain over time, as tau pathology is known to spread through the brain over the course of 4R tauopathy [1]. To test these hypotheses, we employed the PS19 mouse model, which overexpresses the P301S mutant form of the human 4R tau protein under the direction of the mouse prion protein promoter [24]. PS19 mice of both sexes underwent [^18^F]ROStrace imaging at middle (6–11 months old; mo) and late (11+ mo) stages of tau aggregation alongside age-and sex-matched controls. At each timepoint, a subset of the animals was also sacrificed for immunohistochemical examination of tau pathology and oxidative stress. Collectively, these studies revealed progressive elevations in [^18^F]ROStrace signal in PS19 mice compared to age-matched controls, with the most significant signal elevations occurring in brain regions with abundant tau pathology (primarily brainstem, midbrain, and hippocampus). In PS19 brains, elevations in [^18^F]ROStrace signal were also accompanied by progressive increases in nitrotyrosine, an established fluorescent indicator of oxidative stress [25,26,27,28]. Furthermore, tau pathology consistently colocalized with both nitrotyrosine and dihydroethidium (DHE) in PS19 brain samples, independent of the brain region or the extent of pathological burden. These findings provide novel evidence linking 4R tau aggregation with oxidative stress, and indicate that [^18^F]ROStrace holds promise as a means of detecting and monitoring tau-related oxidative stress in living animals and humans.

## 2. Results

### 2.1. PS19 Mice Show Progressive Increases in Brain [^18^F]ROStrace Signal

**Figure 1:**
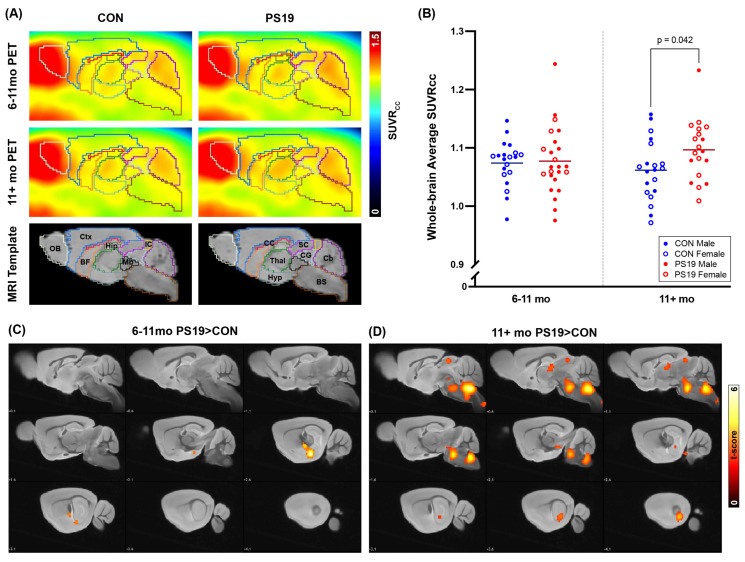
Aged PS19 mice show increased [^18^F]ROStrace signal in brain compared to healthy controls. (**A**) Average [^18^F]ROStrace PET images from control and PS19 mice at 6–11 mo and 11+ mo. Anatomical volumes of interest are displayed as colored outlines on the PET images, with corresponding labels shown on the generic mouse MRIs in the third row. Abbreviations are as follows: OB = olfactory bulb, Ctx = cortex, BF = basal forebrain and septum, CC = corpus callosum, Thal = thalamus, Hyp = hypothalamus, Hip = hippocampus, Mb = midbrain, SC = superior colliculi, IC = inferior colliculi, CG = central grey, BS = brainstem, Cb = cerebellum. (**B**) Whole-brain average SUVRcc values at each timepoint. Here, each dot represents an individual [^18^F]ROStrace scan, with filled dots indicating male animals and outlined dots indicating female animals. As male/female differences were not found to be significant within each genotype (*p* = 0.526 for CON and 0.518 for PS19 by one-way ANOVA), sexes were combined for all subsequent analyses. At each timepoint, horizontal lines indicate group means (6–11 mo CON mean = 1.074; 6–11 mo PS19 mean = 1.077; 11+ mo CON mean = 1.062; 11+ mo PS19 mean = 1.097). At 11+ mo, PS19 mice showed higher average SUVRcc than age-matched controls (*p* = 0.042), as calculated by two-tailed unpaired *t*-test. (**C**,**D**) Statistical parametric maps showing voxels with significantly higher [^18^F]ROStrace signal in PS19 mice at each timepoint. Here, hotter colors indicate higher *t* scores (i.e., lower *p* values), and significant clusters are overlayed onto a generic mouse MRI.

To determine whether 4R tau aggregation is associated with increased oxidative stress in the brains of living PS19 mice, we performed dynamic [^18^F]ROStrace imaging experiments on 6–11 mo and 11–15 mo PS19 and B6C3 (control) animals of both sexes (sample sizes shown in Table S1). Following imaging, we first normalized each animal’s [^18^F]ROStrace PET image to the average signal in that animal’s corpus callosum (CC), as CC was found to be the brain region least affected by tau aggregation and tau-associated oxidative stress in this model (Figure S1). This normalization resulted in a final PET outcome measure of Standardized Uptake Value Relative to CC (SUVRcc). Since previous studies have shown that [^18^F]ROStrace retention in brain remains relatively stable 40+ min after injection [20,21], we next compared the average SUVRcc from 40–60 min in PS19 and B6C3 brains in both age groups (Figure 1). These comparisons revealed no difference in whole-brain average [^18^F]ROStrace signal in 6–11 mo animals; however, aged PS19 mice (11+ mo) showed significantly higher whole-brain average [^18^F]ROStrace signal than aged B6C3 mice (Figure 1A,B; *p* = 0.042). To characterize the spatial distribution of these PS19-specific elevations in [^18^F]ROStrace signal, we next performed voxel-wise statistical comparisons between the PS19 and B6C3 image sets using Statistical Parametric Mapping (SPM) version 12 (Wellcome Centre for Human Neuroimaging, UCL Queen Square Institute of Neurology, London, UK). In 6–11 mo animals, these SPM comparisons revealed small clusters of [^18^F]ROStrace signal elevations in the lateral hippocampi of PS19 mice (Figure 1C), consistent with previously published results demonstrating hippocampal tau pathology in 6–11 mo PS19 animals [24]. In 11+ mo animals, the hippocampus continued to show PS19-specific elevations in [^18^F]ROStrace signal (Figure 1D); however, additional clusters of signal elevations were also found in brainstem, midbrain, and some sections of the mediodorsal thalamus. Notably, previous PET imaging studies in transgenic P301S mice have also found significant elevations in tau burden in brainstem, midbrain, and thalamus [29], supporting the idea that tau aggregation is associated with increased [^18^F]ROStrace signal at both the whole-brain and regional levels.

**Figure 2:**
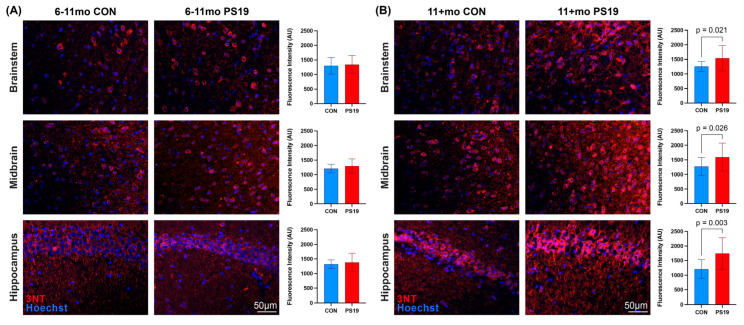
PS19 mice show progressive increases in oxidative damage relative to healthy controls. (**A**) Representative immunofluorescent images showing 3-nitrotyrosine (3NT) staining in brainstem, midbrain, and hippocampus of 6–11 mo control (left) and PS19 (middle) mice. In all images, 3NT staining (red) shows evidence of oxidative stress, while Hoechst staining (blue) shows cell nuclei. At the 6–11 mo timepoint, none of the examined regions showed differences in 3NT fluorescence as calculated by unpaired *t*-tests (right). (**B**) Equivalent images and graphs from 11+ mo animals. Here, every examined region showed higher 3NT fluorescence in PS19 animals compared to healthy controls. For quantification, 5 animals were used per genotype per timepoint.

To corroborate the in vivo PET results and clarify the distribution and magnitude of oxidative damage in our PS19 animals, we next stained PS19 and B6C3 brain sections with 3-nitrotyrosine (3NT), a well-established marker of oxidative and nitrative damage to proteins [25,26,27,28]. This staining revealed no significant differences in 3NT fluorescence between 6–11 mo PS19 and B6C3 brain samples (Figure 2A; *p* values ranging from 0.194–0.650 by two-tailed unpaired *t*-tests), consistent with the [^18^F]ROStrace PET results at this timepoint. By contrast, 11+ mo PS19 tissue samples showed increased 3NT fluorescence in brainstem, hippocampus, and some regions of midbrain (Figure 2B), as expected based on the SPM results shown in Figure 1D. Notably, 11+ mo PS19 animals also showed higher average 3NT fluorescence than 6–11 mo PS19 animals across all regions (*p* < 0.001 via unpaired *t*-test), suggesting that oxidative stress increases with age in these animals. Collectively, both our PET and 3NT staining results indicate that PS19 mice show progressive elevations in oxidative stress in brain over time.

### 2.2. Tau Pathology Becomes Progressively Widespread and Colocalizes with Markers of Oxidative Stress in PS19 Mouse Brain

**Figure 3:**
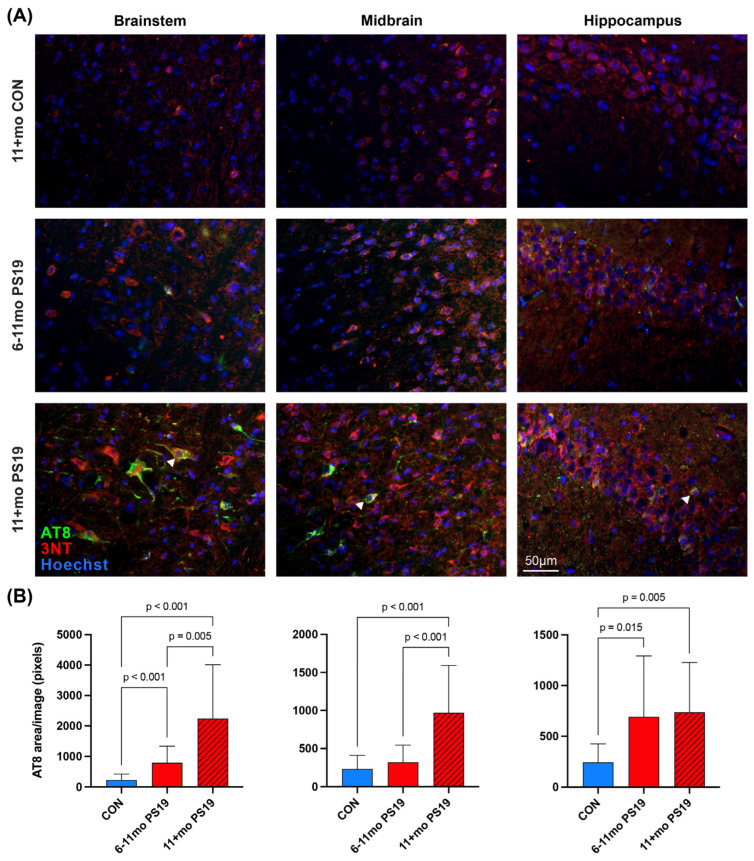
PS19 mice show progressively severe tau pathology in brain regions with significant [^18^F]ROStrace signal elevations. (**A**) Representative immunofluorescent images of control, 6–11 mo PS19, and 11+ mo PS19 brain sections stained with AT8 and 3-nitrotyrosine (3NT). In all images, AT8 staining (green) shows phosphorylated tau pathology, 3NT staining (red) shows evidence of oxidative stress, and Hoechst staining (blue) shows cell nuclei. In 6–11 mo PS19 animals, the hippocampus and brainstem showed the most robust AT8 staining, with most other regions having low AT8 fluorescence. By contrast, the AT8 signal in most 11+ mo animals was robust throughout the brain. Control animals showed minimal AT8 staining at all timepoints. In the bottom row of images (11+ mo PS19), white arrowheads indicate cells that are also shown in Figure 4A. (**B**) Quantification of AT8 pathology in 11+ mo control, 6–11 mo PS19, and 11+ mo PS19 brain samples. Each graph shows the average area of positive AT8 staining per image in each group, and *p* values in all graphs were calculated via Brown-Forsythe and Welch ANOVA tests with follow-up Dunnett’s T3 multiple comparisons test. For quantification, 5 animals were used per genotype per timepoint.

Having demonstrated that PS19 mice show progressive and region-specific increases in both [^18^F]ROStrace signal and 3NT fluorescence, we next sought to characterize the abundance and spatial distribution of tau pathology in PS19 animals at both timepoints. To do this, we stained 6–11 and 11+ mo PS19 and B6C3 brain sections with the phospho-tau antibody AT8 (representative whole-brain image shown in Figure S1A). This staining revealed varying amounts of tau pathology in PS19 animals of all ages, with minimal pathology detected in B6C3 animals regardless of age (Figure 3A). In most 6–11 mo PS19 animals, tau pathology was relatively sparse and predominantly cytosolic, with AT8-positive cells primarily localized to brainstem, midbrain, hypothalamus, superficial cortex, hippocampus, and rarely striatum. Of these regions, the brainstem and hippocampus showed the most abundant AT8 pathology (Figure 3B), although variability among individual animals was high. Interestingly, the AT8 signal in hippocampus was roughly as abundant in 6–11 mo PS19 mice as it was in 11+ mo PS19 mice, supporting the idea that hippocampus is one of the earliest regions to be affected by tau aggregation in the PS19 model. Outside of the hippocampus, 11+ mo PS19 animals showed abundant cytosolic and dendritic tau pathology throughout most of the brain, with brainstem, superior and inferior colliculi, and midbrain showing the highest levels of AT8 fluorescence (bright green puncta in Figure S1A). Notably, this AT8 distribution was similar to the distribution of [^18^F]ROStrace signal elevations in 11+ mo PS19 mice (shown in Figure 1D), supporting the idea that tau pathology is spatially associated with oxidative stress.

While the brainstem, midbrain, and hippocampus showed the most widespread AT8 staining in aged PS19 mice, we also observed less abundant AT8 pathology in thalamus, hypothalamus, cortex, striatum, nucleus accumbens, and grey matter of cerebellum. As in the younger PS19 mice, the AT8 signal varied substantially across 11+ mo animals, with many aged PS19 brains appearing quite similar to those from younger mice. However, a higher proportion of 11+ mo PS19 animals presented with moderate or severe tau pathology (7 out of 17 animals, ~41%) compared to 6–11 mo animals (1 out of 6 animals, ~16%), as expected based on the progressive nature of tau accumulation in this model [24]. At both timepoints, tau pathology was predominantly found in neurons and oligodendrocytes, with minimal AT8 signal observed in microglia or astrocytes. Collectively, our AT8 staining results indicate that PS19 mice develop progressively severe tau pathology in brain as they age, with brainstem, midbrain, and hippocampus showing the most abundant pathology.

**Figure 4:**
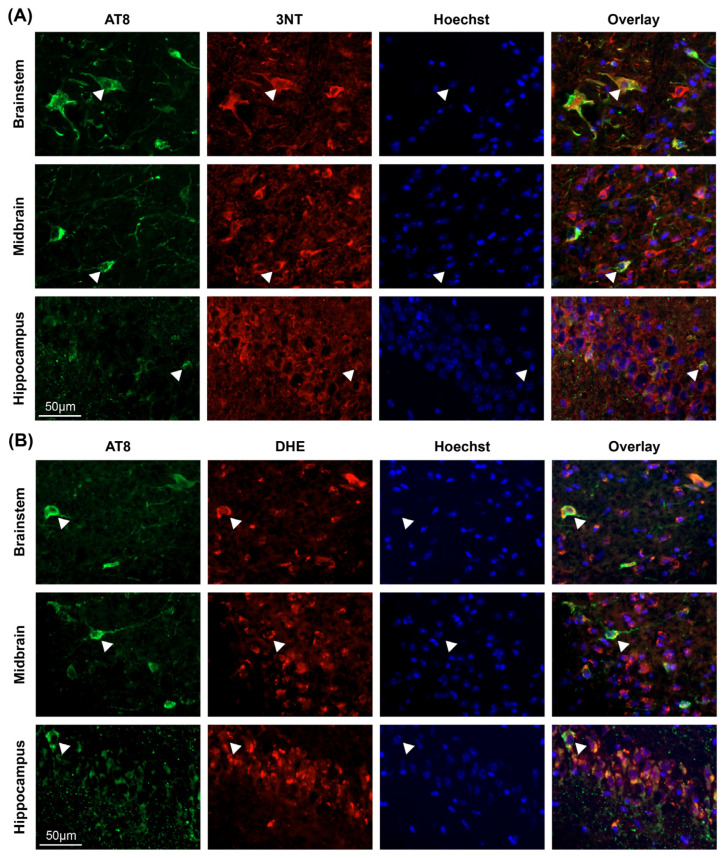
Tau pathology colocalizes with evidence of oxidative stress in PS19 mouse brain. (**A**) Representative immunofluorescent images showing AT8, 3NT, and Hoechst staining in brainstem, midbrain, and hippocampus of 11+ mo PS19 brain samples. In each row, individual channels are shown in separate columns, with the far-right column showing an overlay of all 3 channels. White arrowheads in each image show examples of colocalization between AT8 and 3NT; these cells are also highlighted in Figure 3A. Across all images, 76–86% of the AT8 fluorescence was found to colocalize with 3NT fluorescence. (**B**) Equivalent images showing colocalization between AT8 pathology and dihydroethidium (DHE) in 11+ mo PS19 brain samples. Across these images, 81–85% of the AT8 fluorescence was found to colocalize with DHE fluorescence.

Given the observed similarity between the distributions of tau pathology and [^18^F]ROStrace signal elevations in aged PS19 mice, we next asked whether AT8 staining colocalizes with evidence of oxidative stress at the cellular level. To address this question, we first co-stained PS19 brain sections with AT8 and 3NT (Figure 4A). This staining revealed widespread colocalization between AT8 and 3NT in PS19 brain sections, regardless of timepoint or brain region (examples indicated by white arrowheads in Figure 4A). In the brain regions with the most abundant AT8 pathology (midbrain, hippocampus, and brainstem), 76–86% of the AT8 staining colocalized with 3NT staining (76% in midbrain, 77% in hippocampus, 86% in brainstem), with the remaining 14–24% of the AT8 signal primarily being found in neuronal axons or dendrites. Similar results were observed in DHE-treated PS19 brain tissue samples (Figure 4B), with 81–85% of the AT8 signal colocalizing with DHE fluorescence (81% in midbrain, 83% in brainstem, and 85% in hippocampus). Once again, the remaining 15–19% of the AT8 signal was predominantly localized to neuronal processes, whereas DHE fluorescence was almost exclusively observed in cell bodies (examples indicated by arrowheads in Figure 4B). Collectively, these staining results support the in vivo PET results and indicate that tau pathology is spatially associated with markers of oxidative stress in PS19 mouse brain.

## 3. Discussion

Oxidative stress is thought to be an early and central contributor to the pathogenesis of PSP, CBD, and other 4R tauopathies [3,4,5,6,7,8,9,10,11,12,13,14,15,16,17,18], yet we currently lack reliable methods for detecting and tracking tau-associated oxidative stress in vivo. In this study, we demonstrate that PET imaging with the ROS-sensitive radiotracer [^18^F]ROStrace is capable of detecting increased oxidative stress in the brains of living PS19 mice, which show 4R tau aggregation similar to that seen in human tauopathy. PS19-specific elevations in [^18^F]ROStrace signal were first detectable in the hippocampus of 6–11 mo animals and subsequently spread to the midbrain, brainstem, and thalamus of 11+ mo animals (Figure 1). The distribution of [^18^F]ROStrace signal elevations in 11+ mo PS19 animals aligned well with that of phosphorylated tau pathology in these animals (Figure S1A) and also aligned with previously published distributions of tau pathology in PS19 mice [29]. The [^18^F]ROStrace PET results were substantiated by 3-nitrotyrosine staining (Figure 2), which showed significant and progressive increases in 3NT fluorescence in brain regions with elevated [^18^F]ROStrace signal. PS19-specific increases in [^18^F]ROStrace signal were also accompanied by progressive increases in AT8 staining (Figure 3), with the hippocampus in particular showing an early rise in both AT8 fluorescence (Figure 3B) and [^18^F]ROStrace signal (Figure 1C). Moreover, AT8 pathology consistently colocalized with both 3NT and DHE in PS19 brain samples (Figure 4), indicating that tau aggregation is spatially associated with oxidative stress at the cellular level. Collectively, our results indicate that 4R tau aggregation is associated with increased oxidative stress in the brains of PS19 mice, and highlight the potential of [^18^F]ROStrace as a means of investigating tau-associated oxidative stress noninvasively.

Although tau aggregation has previously been linked with oxidative stress in both human patients [3,4,6,7,8,9,10,30] and animal models [11,12,13], it is well-established that numerous psychological and physiological processes are robustly associated with increased ROS production [31,32]. Thus, a central challenge associated with the in vivo detection of 4R tau-associated oxidative stress is differentiating between ‘disease-associated’ increases in ROS production (i.e., those related to 4R tau aggregation) and ‘nonspecific’ increases (those unrelated to tau aggregation). In this study, we addressed this challenge by normalizing each [^18^F]ROStrace PET scan to the average signal in the corpus callosum, a major white matter tract that consistently showed low levels of both tau pathology and histological markers of oxidative stress (Figure S1). Normalization of the PET data in this manner improved the sensitivity of the imaging to tau-associated oxidative stress specifically, and also helped to control for animal-to-animal differences in tracer metabolism and pharmacokinetics. Indeed, normalization of [^18^F]ROStrace imaging data has proven to be critical for the successful detection of disease-associated oxidative stress in multiple other mouse models of proteinopathy [21,22], since non-normalized [^18^F]ROStrace results consistently show high levels of variability even amongst healthy controls [21]. Notably, substantial variability was also observed in our PS19 animals themselves, with some individuals showing robust phospho-tau pathology at ~9 mo and others showing minimal pathology at ~16 mo. Such variability is an established characteristic of this model [24,33,34,35,36,37], and similar patterns have also been observed in other widely-used mouse models of proteinopathy [22]. As such, future studies using the PS19 line may benefit from categorizing animals based on symptom severity or pathological burden rather than age, as individual timepoints (e.g., 6, 9, or 11 mo) are likely to encompass PS19 animals in a wide range of disease states.

Although most PS19 mice used in this study did eventually develop the widespread tau pathology that is characteristic of this model, it is worth noting that our PS19 animals generally showed a delayed disease phenotype relative to that described in some early studies using this mouse line. For example, Yoshiyama et al. reported in 2007 that ~80% of their PS19 animals died before the age of 12 mo, with a median survival time of ~9 months [24]. By contrast, none of the PS19 animals in our colony died before their predetermined endpoints (age range = 9–16 mo, median ~13 mo) and a substantial number of our PS19 mice (*n* = 10) survived past the age of 16 mo. Likewise, widespread tau pathology has previously been noted in PS19 animals as early as 6 mo [24], yet we did not observe positive AT8 staining in our PS19 mice until ~9 mo or older. Importantly, our current findings are consistent with more recent studies in the PS19 model, which report that both neurodegeneration and hyperphosphorylated tau deposition now occur later in the PS19 lifespan than originally reported by Yoshiyama et al. For example, Zhang et al. reported in 2012 that significant neural death in PS19 mice occurred at ~12 mo rather than ~9 mo [33], and Iba et al. found in 2013 that phospho-tau pathology in PS19 animals became detectable at >12 mo rather than ~6 mo [34]. Thus, our current results support the idea that phenotypic drift has led to a notable delay in disease progression in PS19 animals compared to initial reports, although it is unclear whether this pattern extends to all PS19 animals or is unique to animals bred at the University of Pennsylvania. Regardless, stereotaxic injections of preformed tau fibrils (PFFs) into the brains of young PS19 animals have been shown to both accelerate disease progression and reduce inter-animal variability in the onset and distribution of tau pathology [34]. Therefore, PFF-injected PS19 mice may represent a more consistent and reliable model system for future investigations into the bidirectional relationship between tau aggregation and oxidative stress.

While the results described in this study clearly indicate that 4R tau aggregation is associated with oxidative stress in PS19 mouse brain, the specific mechanisms linking tau aggregation and increased ROS production remain incompletely understood. For this reason, it is notable that we observed significant increases in 3NT fluorescence in aged PS19 animals, as these results suggest that increased levels of ROS in vivo lead to increased nitration of tau proteins by ROS and reactive nitrogen species (RNS) such as peroxynitrite. Indeed, previous studies have demonstrated that peroxynitrite-mediated nitration of tau accelerates the formation of neurotoxic tau inclusions [16,18], and abundant evidence supports the idea that peroxynitrite specifically is an early and central contributor to protein aggregation across the proteinopathies [28,38,39,40,41,42]. Thus, it appears likely that the nitrated tyrosine residues labeled in Figure 2, Figure 3 and Figure 4 are not simply byproducts of tau-associated oxidative and nitrative stress, but are also disease-relevant protein modifications that actively accelerate and perpetuate tau aggregation in PS19 animals. Although this idea was not a specific focus of the current study, antioxidant drugs like thiamine and methylene blue have shown promise in ameliorating peroxynitrite-induced oxidative damage [43,44], and future studies in PS19 animals could therefore seek to determine whether such antioxidants show efficacy in reducing tau-associated increases in [^18^F]ROStrace signal and/or 3NT fluorescence.

While this study did not seek to establish the specific biological sources of tau-associated ROS in PS19 animals, previous experiments in the PS19 line have demonstrated that microglial and astrocytic activation precede tau tangle formation by several months [24]. Since activated microglia [45,46,47,48] and reactive astrocytes [49,50,51,52] are both thought to be major sources of ROS and RNS in human 4R tauopathies, gliosis stands out as one potential contributor to the increased [^18^F]ROStrace signal observed in our PS19 animals. Likewise, mitochondria are known to be significant sources of ROS within the CNS even under healthy conditions [53,54], and tau oligomers and aggregates have been shown to induce mitochondrial dysfunction and increase mitochondrial ROS production [55,56,57]. Moreover, we have found that silencing mitochondrial ROS production is sufficient to preserve cognition and prevent pathology in triple transgenic mice harboring the same P301S tau mutation [58]. Therefore, mitochondrial dysfunction stands out as another likely contributor to the observed tau-specific increases in oxidative stress.

Because multiple ROS-generating mechanisms likely contribute to [^18^F]ROStrace signal elevations in PS19 mice, future [^18^F]ROStrace imaging studies should now seek to attenuate these signal increases via mechanistically distinct pharmacological interventions. For example, several mitochondria-targeted antioxidants have shown efficacy in reducing oxidative stress in rodent tauopathy models, including MitoQ [58], MitoSOD [59], and the SkQ family of compounds [60,61,62], among others. Similar drugs are available for microglia [48,63,64,65] and astrocytes [66,67,68], and multiple other pharmaceuticals have also been shown to ameliorate tau-associated neurotoxicity in PS19 mice, including the microtubule-stabilizing agents epothilone D [33] and dictyostatin [69], the tau aggregation inhibitor anle138b [70], and the toll-like receptor 2 antagonist wtTIDM [71], among others [72]. Since each of these drugs target different components of the tau-associated neuroinflammatory cascade, such interventional studies could provide valuable insights into the specific biochemical processes underlying the [^18^F]ROStrace signal in PS19 mice. Indeed, [^18^F]ROStrace PET is uniquely positioned to enhance the study of antioxidant and anti-inflammatory interventions in PSP, CBD, and other 4R tauopathies, as it enables the longitudinal assessment of oxidative burden in living subjects at multiple stages of disease progression and treatment. While the current study primarily aimed to compare the in vivo [^18^F]ROStrace PET results with postmortem staining results, longitudinal assessments could enable us to track individual subjects over time, offering deeper insights into how oxidative stress evolves and progresses over the course of neurodegenerative diseases. Additionally, it is essential to recognize that PET-based biomarkers like [^18^F]ROStrace can serve as both diagnostic tools and pathological markers. By understanding how oxidative stress correlates with disease advancement at various stages, we could refine the use of these biomarkers for not only detecting disease presence but also for monitoring its progression and response to therapeutic interventions.

## 4. Materials and Methods

### 4.1. Animals

The PS19 and B6C3 animals used for this study were generously donated by Drs. Hong Xu and Virginia Lee at the University of Pennsylvania. Equivalent animals may be purchased from the Jackson Laboratory (Bar Harbor, ME, USA; strain numbers 008169 (PS19) and 100010 (B6C3)). All animal procedures complied with the NIH Guide for the Care and Use of Experimental Animals and were approved by the Animal Care and Use Committee at the University of Pennsylvania.

### 4.2. Preparation of [^18^F]ROStrace

The [^18^F]ROStrace doses used for these studies were prepared as previously described [20]. The tracer was synthesized using a Trasis AllInOne radiochemistry synthesizer (Liège, Belgium), and the concentrated product was diluted with 0.6 mL of ethanol, 0.1% ascorbic acid, and 6 mL of sterile saline. The final product was then filtered through a 0.2 μM nylon filter prior to injection. The radiochemical yield was 4–20%, and the specific activity was 74 GBq/mol.

### 4.3. Neuroimaging

PET and CT imaging for this project was performed using a Molecubes β-Cube PET scanner and X-Cube CT scanner (Molecubes, Ghent, Belgium). Prior to each scan, animals were anesthetized using 1.5–2.5% isoflurane, and a needle with attached catheter was inserted into the lateral tail vein and taped in place. To make the tail vein catheters, we used 30ga needles from Braintree Scientific (Braintree, MA, USA, item NS 30G) and 0.011″ × 0.024″ polyethylene tubing (item PE10 from Braintree Scientific). Once the tail vein catheters were successfully inserted, the animals were transferred to heated beds in the PET scanner and subsequently injected with 200–300 μCi of [^18^F]ROStrace. Following tracer injection, dynamic [^18^F]ROStrace PET images were acquired for 60 min, with a ~5 min CT acquisition following each PET scan. Once imaging was complete, all PET and CT images were reconstructed using manufacturer-supplied reconstruction software.

### 4.4. PET Image Analysis

PET and CT images were analyzed using Pmod version 3.7 (PMOD Technologies Ltd., Zurich, Switzerland). Dynamic [^18^F]ROStrace PET images were first coregistered to their corresponding CT images using Pmod’s rigid-body alignment tool. Once the PET-CT alignment was checked, each CT image was manually scaled, rotated, and translated so that it aligned with the M. Mirrione mouse brain atlas [73]. The resulting transformation was then applied to the corresponding PET image to align it with both the CT image and the brain template. Following realignment, PET images were normalized based on the applicable injected dose and body weight to convert them to standardized uptake value (SUV) units. Finally, a modified version of the M. Mirrione mouse brain atlas [22] was used to calculate the average SUV in the corpus callosum, and the SUV PET results were divided by this number to convert each image to SUVRcc units.

Once all SUVRcc images had been generated, voxel-level statistical comparisons were performed using SPM version 12 (Wellcome Centre for Human Neuroimaging, UCL Queen Square Institute of Neurology, London, UK) with the SPMMouse toolbox [74] implemented in MATLAB R2017b (MathWorks Inc., Natick, MA, USA).

### 4.5. Immunohistochemistry and Microscopy

Once all applicable imaging experiments were complete, animals were anesthetized with 2–4% isoflurane and transcardially perfused with chilled heparinized saline. Following perfusion, the brains were extracted and cut in half, and individual hemispheres were drop-fixed in 4% paraformaldehyde for 2–5 days at 4 °C. Fixed hemispheres were then transferred to 30% sucrose for cryoprotection and embedded in Tissue-Tek optimal cutting temperature compound (Sakura Finetek Japan Co., Ltd., Tokyo, Japan). Once the hemispheres were frozen (performed in a slurry of dry ice and isopentane) and sectioned, 12 μm thick brain slices were mounted on slides (Fisherbrand Superfrost Plus Microscope Slides, Fisher Scientific, Hampton, NH, USA) for IHC. Slides were first warmed to room temperature and rinsed with phosphate-buffered saline (PBS), before being permeabilized with a 0.5% solution of Triton X-100 in PBS. Blocking was accomplished via a 10 min incubation with 100 mM Glycine plus a 1 h incubation with 10% bovine serum albumin (BSA; note that this was found to work well for both mouse and rabbit primary antibodies). Following blocking, 1% BSA was used to rinse the slides, which were then incubated with primary antibodies (shown in Table S2) overnight at 4 °C. The next day, slides were rinsed with 1% BSA before being incubated with the applicable secondary antibodies for 1 h at room temperature. When co-staining was required, slides were treated with an additional primary/secondary antibody combination (from a different host species) for 1 h at room temperature with 1% BSA washes in between. Finally, all slides were incubated for 1 min with the nuclear stain Hoechst (1:2000 in PBS) and mounted with VectaShield Antifade Mounting Media (Vector Laboratories, Newark, CA, USA, stock no. 101098-042).

Following staining, most slides were imaged using a Zeiss Axio Observer Microscope (Carl Zeiss AG, Oberkochen, Germany). In some cases, a Keyence BZ-X810 Microscope (Keyence Corporation, Osaka, Japan) was also used to acquire panoramic whole-brain images. Confocal images were acquired using an Andor BC43 CF Benchtop Confocal Microscope (Oxford Instruments, Abingdon, UK). For quantification of 3NT, AT8, and DHE staining, 5 animals were imaged per genotype per timepoint, and 4 20× images were acquired per region per animal using the Zeiss microscope. Red, green, and blue channels were acquired in all images used for quantification, and antibody-specific Fiji scripts were used to quantify the positive staining and/or colocalization in each image (Fiji version 2.14.0/1.54f). All applicable Fiji scripts are available at the following GitHub repository: https://github.com/Evangall/fiji-scripts (updated on 27 August 2024). For quantification, all acquisition parameters were kept identical across groups being compared.

### 4.6. Dihydroethidium (DHE) Injections

Prior to DHE injections, all animals were weighed, and DHE solid (dose = 20 mg/kg) was dissolved in DMSO (10 µL DMSO/mg DHE) under low-light conditions. The DHE/DMSO solution was then diluted to 1 mg/mL using ethanol, Tween 20, and sterile saline [22] and subsequently injected into the animals I.P. under low-light conditions. Two hours after injection, the animals were sacrificed, perfused with PBS and 4% paraformaldehyde, and dissected (also performed under low-light conditions). The resulting brain hemispheres were drop-fixed in 4% paraformaldehyde for 2 h, submerged in 10% sucrose overnight, and finally embedded and sectioned as described in Section 4.5. Following sectioning, slides were rinsed with PBS, counterstained with Hoechst (1:2000) for 1 min, and immediately mounted and imaged using the Zeiss or Keyence microscopes. When co-staining with DHE and another antibody was required, we first warmed and rinsed the slides as described above. Subsequently, we permeabilized the slides with 0.5% triton for 15 min, blocked them with 100 mM glycine for 10 min, and treated them with the applicable primary and secondary antibodies for 1 h each at room temperature. We then stained them with Hoechst (1:2000) for 1 min, mounted, and imaged them as described above.

## 5. Conclusions

This study demonstrates that PS19 mice—which show tau aggregation similar to that seen in human 4R tauopathy—show significant and progressive increases in brain [^18^F]ROStrace signal relative to age-matched control mice. [^18^F]ROStrace signal elevations were most pronounced in brain regions bearing abundant tau pathology, and tau aggregates colocalized with multiple established histological indicators of oxidative stress. To our knowledge, this study represents the first use of PET imaging to detect tau-associated oxidative stress in the living brain. As such, these results provide novel evidence that tau aggregation is associated with increased oxidative stress, and highlight the utility of [^18^F]ROStrace as a noninvasive means of investigating tau-associated oxidative stress in preclinical models of 4R tauopathy.

## Data Availability

The Fiji scripts used for this project are available at https://github.com/Evangall/fiji-scripts (updated on 27 August 2024). All other data and materials will be made available by the authors upon request.

## Supplementary Materials

The following supporting information can be downloaded at: https://www.mdpi.com/article/10.3390/ijms26051845/s1.

## Abbreviations

3NT	3-nitrotyrosine
4R tau	4-repeat tau (tau isoforms containing 4 microtubule-binding domains)
AD	Alzheimer’s disease
AGD	argyrophilic grain disease
aSyn	alpha-synuclein
CBD	corticobasal degeneration
CC	corpus callosum
CTE	chronic traumatic encephalopathy
DHE	dihydroethidium
GFAP	glial fibrillary acidic protein
Iba1	ionized calcium binding adaptor molecule 1
IHC	immunohistochemistry
mo	month-old
PET	positron emission tomography
PFF	preformed fibril
PSP	progressive supranuclear palsy
RNS	reactive nitrogen species
ROS	reactive oxygen species
SPM	statistical parametric mapping
SUV	standardized uptake value
SUVRcc	standardized uptake value relative to corpus callosum
