# Supplementary material for: Noninvasive Detection of Oxidative Stress in a Mouse Model of 4R Tauopathy via Positron Emission Tomography with [18F]ROStrace
Source: Int J Mol Sci. 2025 Feb 21;26(5):1845. doi: 10.3390/ijms26051845 (PMC11899037; doi:10.3390/ijms26051845)
Supplement: Supplementary file 1 [file ijms-26-01845-s001.zip › ijms-3469250-supplementary.pdf]

Electronic Supplementary Material

Noninvasive Detection of Oxidative Stress in a Mouse Model of 4R Tauopathy via Positron Emission Tomography with [^{18}F]ROStrace

Journal: International Journal of Molecular Sciences

Evan Gallagher [1,2], Shihong Li [2], Hsiaoju Lee [2], Hong Xu [3]
Virginia M-Y. Lee [3], Robert H. Mach [2], Meagan J. McManus [1]

1. Department of Anesthesia and Critical Care Medicine, Children's Hospital of Philadelphia, Philadelphia, PA, USA
2. Department of Radiology, University of Pennsylvania, Philadelphia, PA, USA
3. Center for Neurodegenerative Disease Research, University of Pennsylvania, Philadelphia, PA, USA

Corresponding author:
Meagan J. McManus
Leonard and Madlyn Abramson Pediatric Research Center, Rm 403
3615 Civic Center Blvd
Philadelphia, PA, USA, 19104
Email: mcmanusm@chop.edu

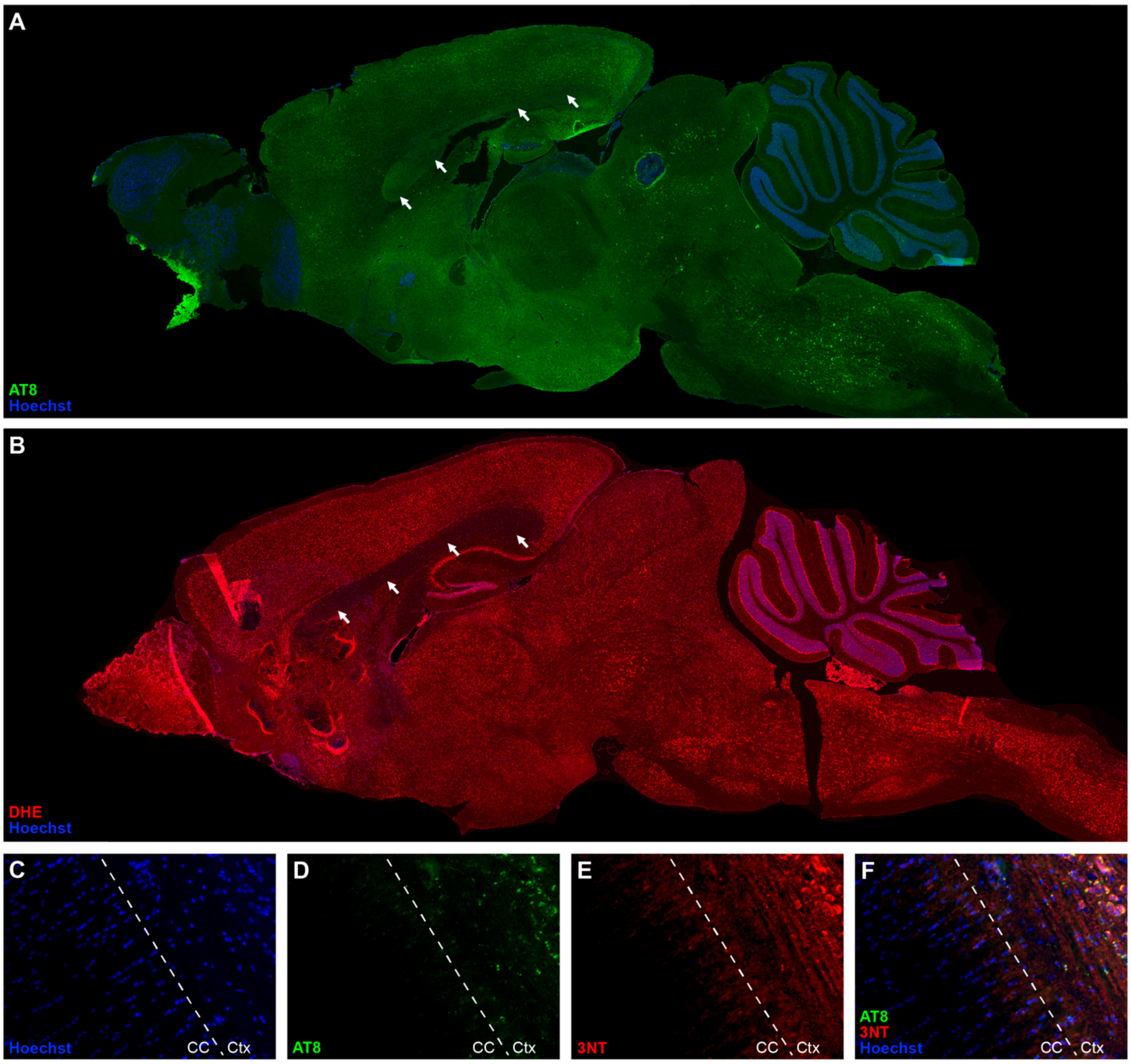

Supplementary Figure S1. Corpus callosum (CC) shows minimal evidence of tau aggregation and tau-associated oxidative stress in PS19 mouse brain. **(a)** Whole-brain immunofluorescent image showing phosphorylated tau pathology (AT8; green puncta) in a 13mo PS19 brain section. While AT8 staining was widespread throughout most of the brain, minimal AT8 pathology was observed in the CC (indicated by white arrows). **(b)** Whole-brain fluorescent image showing the distribution of the ROS-sensitive dye dihydroethidium (DHE; red) in a 13mo PS19 brain section. Again, minimal DHE signal was observed in CC. **(c-f)** Representative immunofluorescent images showing cell nuclei (Hoechst; blue), tau pathology (AT8; green), and nitrated tyrosine residues in proteins (3NT; red) in the CC and cortex (Ctx) of a 12mo PS19 mouse. In these images, a dashed white line indicates the approximate border between the CC and the Ctx. Notably, while AT8 colocalized with 3NT throughout the Ctx (F), minimal AT8 fluorescence was noted in CC (D), suggesting that CC is largely spared by phosphorylated tau pathology in the PS19 model.

	Total		Female		Male	
	**6-11 mo**	**11+ mo**	6-11 mo	11+ mo	6-11 mo	11+ mo
**CON**	**20**	**20**	8	11	12	9
**PS19**	**24**	**21**	7	12	17	9

Supplementary Table S1. Sample sizes for PET imaging experiments.

	Name	Host	Target	Company	Product Number	Primary Dilution (if applicable)	Secondary Dilution (if applicable)
Primary	AT8	Mouse	Phosphorylated tau	ThermoFisher	MN1020	1:200	1:300
	3NT	Rabbit	Nitrated tyrosine residues	MilliporeSigma	AB5411	1:200	1:300
	Iba1	Rabbit	Microglia	Fujifilm Wako	019-19741	1:200	1:300
	GFAP	Mouse	Astrocytes	MilliporeSigma	MAB360	1:300	1:500
	GFAP	Rabbit	Astrocytes	MilliporeSigma	AB5804	1:200	1:300
	ASPA	Rabbit	Oligodendrocytes	GeneTex	GTX113389	1:200	1:300
	NeuN	Rabbit	Neurons	ThermoFisher	PA5-78499	1:200	1:300
Secondary	Alexa 488	Goat	Anti-rabbit	Abcam	ab150077		
	Alexa 488	Goat	Anti-mouse	Abcam	ab150113		
	Alexa 568	Goat	Anti-rabbit	Abcam	ab175471		
	Alexa 568	Goat	Anti-mouse	Abcam	ab175473		
Other	Hoechst	*n/a*	DNA/cell nuclei	ThermoFisher	H3570	1:2000	
	Dihydroethidium	*n/a*	ROS-sensitive fluorescent dye	MilliporeSigma	37291	20mg/kg, diluted to 1mg/mL	

Supplementary Table S2. Antibodies and fluorescent stains used for this project.
